# The whole-cell proteome shows the characteristics of macrolides-resistant *Bordetella pertussis* in China linked to the biofilm formation

**DOI:** 10.1007/s00203-023-03566-0

**Published:** 2023-05-06

**Authors:** Zhe Lv, Sha Yin, Kaichong Jiang, Wei Wang, Yang Luan, Shuang Wu, Jianfei Shi, Zhe Li, Xiao Ma, Zengguo Wang, Hong Yan

**Affiliations:** 1 Department of Epidemiology and Health Statistics, School of Public Health, Xi’an Jiaotong University Health Science Center, Xi’an, 710061 China; 2 National Regional Children’s Medical Center (Northwest), Key Laboratory of Precision Medicine to Pediatric Diseases of Shaanxi Province, Xi’an Key Laboratory of Children’s Health and Diseases, Shaanxi Institute for Pediatric Diseases; Xi’an Children’s Hospital, Affiliated Children’s Hospital of Xi’an Jiaotong University, No. 69, Xijuyuan Lane, Xi’an, 710003 China; 3 Department of Clinical Laboratory, Xi’an Children’s Hospital, Affiliated Children’s Hospital of Xi’an Jiaotong University, No. 69, Xijuyuan Lane, Xi’an, 710003 China; 4 Xi’an Center for Disease Control and Prevention, 599 Xiying Road, Xi’an, 710054 China; 5 Department of Diphtheria, Tetanus and Pertussis Vaccine and Toxins, National Institute for Food and Drug Control, Beijing, China

**Keywords:** Bordetella pertussis, Biofilm, Whole-cell proteome, Macrolide resistance, Fitness

## Abstract

The macrolides-resistant *Bordetella pertussis* (MR-*Bp*) isolates in China evolved from the *ptxP1/fhaB3* allele and rapidly became predominant, suggestive of an adaptive transmission ability. This was different from the global prevalent *ptxP3* strains, in which MR-*Bp* was rarely reported. The study aimed to determine the underlying mechanism responsible for fitness and resistance in these two strains. We identify proteomic differences between *ptxP1/fhaB3* and *ptxP3/fhaB1* strains using tandem mass tag (TMT)-based proteomics. We then performed in-depth bioinformatic analysis to determine differentially expressed genes (DEGs), followed by gene ontology (GO), and protein–protein interaction (PPI) network analysis. Further parallel reaction monitoring (PRM) analysis confirmed the expression of four target proteins. Finally, the crystal violet method was used to determine biofilm-forming ability. The results showed that the main significantly different proteins between the two represent isolates were related to biofilm formation. Furthermore, we have confirmed that *ptxP1/fhaB3* showed hyperbiofilm formation in comparison with *ptxP3/fhaB1*. It is suggested that the resistance and adaptability of *ptxP1/fhaB3* strains may be related to the formation of biofilm through proteomics. In a word, we determined the significantly different proteins between the *ptxP1/fhaB3* and *ptxP3/fhaB1* strains through whole-cell proteome, which were related to biofilm formation.

**Supplementary Information:**

The online version contains supplementary material available at 10.1007/s00203-023-03566-0.

## Background

*Bordetella pertussis* (*Bp*) is the pathogen of pertussis, a severe respiratory infectious disease. Although pertussis has been well controlled globally through pertussis vaccination, the incidence of pertussis has increased significantly in recent years, showing the characteristics of a “resurgence” (Clark 2014). Around the 1980s, isolates carrying the *ptxP1* allele became the predominant *ptxP* type in many countries (Mosiej et al. 2015). Since early 2000, *PtxP3/fhaB1 Bp* lineage has almost replaced the resident *ptxP1*-*Bp* lineage resulting in an almost worldwide selective sweep (Consortium 2019; Xu et al. 2019a, b). By contrast, the Chinese *ptxP3* allele first emerged in 2000, but unlike trends elsewhere, *ptxP1* remained predominant among the isolates (Wang and He 2015; Xu et al. 2015). Moreover, the macrolides-resistant (MR) *ptxP3/fhaB1 Bp* strains were occasionally reported without further dissemination (Guillot et al. 2012), which may be related to the fitness cost (greatly reduced pathogenicity) of *Bp* after MR.

However, there has become an explosive increase after the emergence of macrolides-resistant B. pertussis (MR-*Bp*) in China in 2011 (Wang et al. 2013, 2014; Liu et al. 2018). From our previous genomic results, we found that all MR-*Bp* strains in China have evolved from *ptxP1/fhaB3* lineage (Xu et al. 2019). Recently, the MR-*Bp* strain in Japan and Vietnam was reported and related to the *ptxP1/fhaB3* lineage, which raises the possibility that MR-*Bp* could spread all over the world (Kamachi et al. 2020; Yamaguchi et al. 2020; Feng et al. 2021). Thus, *ptxP1/fhaB3* MR-*Bp* became the dominant strain in China, which is contrary to the phenomenon of the fitness cost of loss of virulence after macrolides resistance of *Bp* (Weiss and Falkow 1984). However, the mechanism of this phenomenon was under dispute.

Microbial biofilm is an organized aggregate of microorganisms, which is resistant to an extreme environment, such as increasing microbial resistance against various antibiotics (Yin et al. 2019). *Bp* is more resistant to macrolide antibiotics in the biofilm and biofilm spatial structure confers a consistent and robust fitness advantage (Dorji et al. 2016; Deschaine et al. 2018), suggesting that the adaptive transmission ability of *ptxP1/fhaB3* MR-*Bp* may be related to the ability of biofilm formation.

Therefore, to reveal the possible mechanism of the fitness of prevalent MR-*Bp*, mass spectrometry-based proteomics of *ptxP1/fhaB3* MR-*Bp* and the globally prevalent macrolides-sensitive (MS) *ptxP3/fhaB1 Bp* were performed in this study to screen the characteristic proteins of prevalent MR-*Bp* and detect the biofilm-forming ability of the two representative isolates.

## Methods

### Bacterial culture

*Bordetella pertussis* strains 19,147 (*ptxP1/fhaB3*) and 19,068 (*ptxP3/fhaB1*) were collected from the patients suffering from pertussis from China in 2019. These two strains from glycerol stocks were grown on Bordet–Gendou agar plates in parallel and incubated at 37 °C for 3 days. Pure cultures were obtained by sub-culturing a single clone onto second Bordet–Gendou agar plates and incubated again at 37 °C for 2–3 days. A loopful of pure colonies was inoculated into 40 ml of Stainer–Scholte (SS) with 1% Heptakis ((2,6-o-dimethyl) β-cyclodextrin) and incubated for 24 h with shaking at 37 °C with the starting OD600 adjusted to 0.1.

### Sample preparation

After incubation, the whole-cell and supernatant were separated by centrifugation at 3000 × g at 4 °C for 15 min. The whole-cell pellet was washed three times with phosphate-buffered saline (PBS) and then frozen quickly with liquid nitrogen. The whole-cell pellet is stored at − 80 °C for subsequent tandem mass tag (TMT)-based proteomics analysis.

### TMT-based proteomics analysis

The pellet was lysed with SDT (4% (w/v) SDS, 100 mM Tris/HCl pH7.6, 0.1 M DTT) and the number of proteins was quantified with the BCA Protein Assay Kit (Bio-Rad, USA). Subsequently, the proteins were digested with trypsin to obtain peptides (Chou and Schwartz 2011), which were further desalted by C18 Cartridge. After lyophilization, the peptides were reconstituted with 40 μL 0.1% formic acid solution and were quantified (OD280). TMT reagent (Thermo Fisher Scientific) was used to label the resulting 100 μg peptides, according to the manufacturer’s instructions. Labeled peptides were fractionated by the High pH Reversed-Phase Peptide Fractionation Kit (Thermo Scientific). Then, the samples were separated using an EASY-nLC liquid chromatography instrument (Thermo Fisher Scientific) and LC–MS/MS analysis was performed on a Q-Exactive mass spectrometer (Thermo Scientific). The protein identification and quantification were accomplished using Mascot2.2 and Proteome Discoverer1.4. We aligned the acquired protein sequence against the *Bordetella pertussis* sequence from Protein Sequence Database (Uniprot database) and the protein ratios were obtained by taking the median ratio of peptides that are unique for a protein group. Three biological replicates were performed for each strain.

### Bioinformatics analysis

CELLO (http://cello.life.nctu.edu.tw/), which is a multi-class SVM classification system, was used to predict protein subcellular localization. The protein sequences of the selected differentially expressed proteins were locally searched using the NCBI BLAST + client software (NCBI-blast-2.2.28 + -win32.exe) and InterProScan to find homolog sequences, and then, gene ontology (GO) terms were mapped and sequences were annotated using the software program Blast2GO. The Fisher’s exact test is used to compare the distribution of each GO classification in the protein set and perform the enrichment analysis of GO annotations on the target protein set. Up-regulated and down-regulated proteins were defined as having fold changes (FC) > 1.2 and < 0.82, respectively. A two-tail Student’s t test was then performed with *p* < 0.05 assigned as significant. Three biological replicates were used for each strain. To explore the role of the functional link between different proteins of the two strains, we performed protein–protein interaction (PPI) network on the significantly different proteins. The PPI information of the studied proteins was based on the STRING database (http://string-db.org/) and Cytoscape software (version 3.8.2). The GO annotation results and volcano results were plotted by R scripts.

### Parallel reaction monitoring (PRM)-based protein quantification

PRM is a liquid chromatography–mass spectrometry-based targeted peptide/protein quantification method. Proteins that were identified to be significantly different in TMT experiments were confirmed using PRM measurements. The proteins of whole-cell were trypsin digested, a liquid chromatography separation systems’ (HPLC) system was used for chromatographic separation, and Q-Exactive HF mass spectrometer (Thermo Scientific) was used for mass spectrometry analysis. Here, we selected 11 node proteins in the PPI network (Cpn10, RpoZ, RpsC, HscA, RpoD, RplB, RpsD, prn, fim2, fim3, and fimD) for PRM verification, of which four target proteins can monitor credible peptides, namely Prn, Fim2, Fim3, and RpoD. The list of target peptides and sub-ions for PRM quantification is shown in Supplementary Table 1.

### Biofilm formation assay

To determine the biofilm formation of 19,147 (*ptxP1/fhaB3*) and 19,068 (*ptxP3/fhaB1*), the strains were grown for 4 days at 37 ℃ and stained with crystal violet every 24 h. The specific steps were as follows. *Bp* from overnight SS liquid cultures was diluted to OD600 = 0.1. 100 ul Bp culture were added to 96-well plates and incubated for 96 h at 37℃ at rest. The culture medium was discarded every 24 h and the cells were washed three times with PBS, supplemented with fresh culture medium. After incubation, 96-well plates were washed three times with PBS and left to dry. Crystal violet (1% w/v, 150 μL) was added to each well and the plate was incubated for 10 min at room temperature. The supernatant was then discarded and 200 µL of ethanol containing 10% acetic acid was added. After 10 min of incubation at room temperature, biofilm formation was then visualized by measuring the OD600. Five biological replicates were used at each time point. All statistical analyses were completed using GraphPad Prism 8.0.

## Results

### Overview of the significantly different proteins in whole-cell

**Fig. 1:**
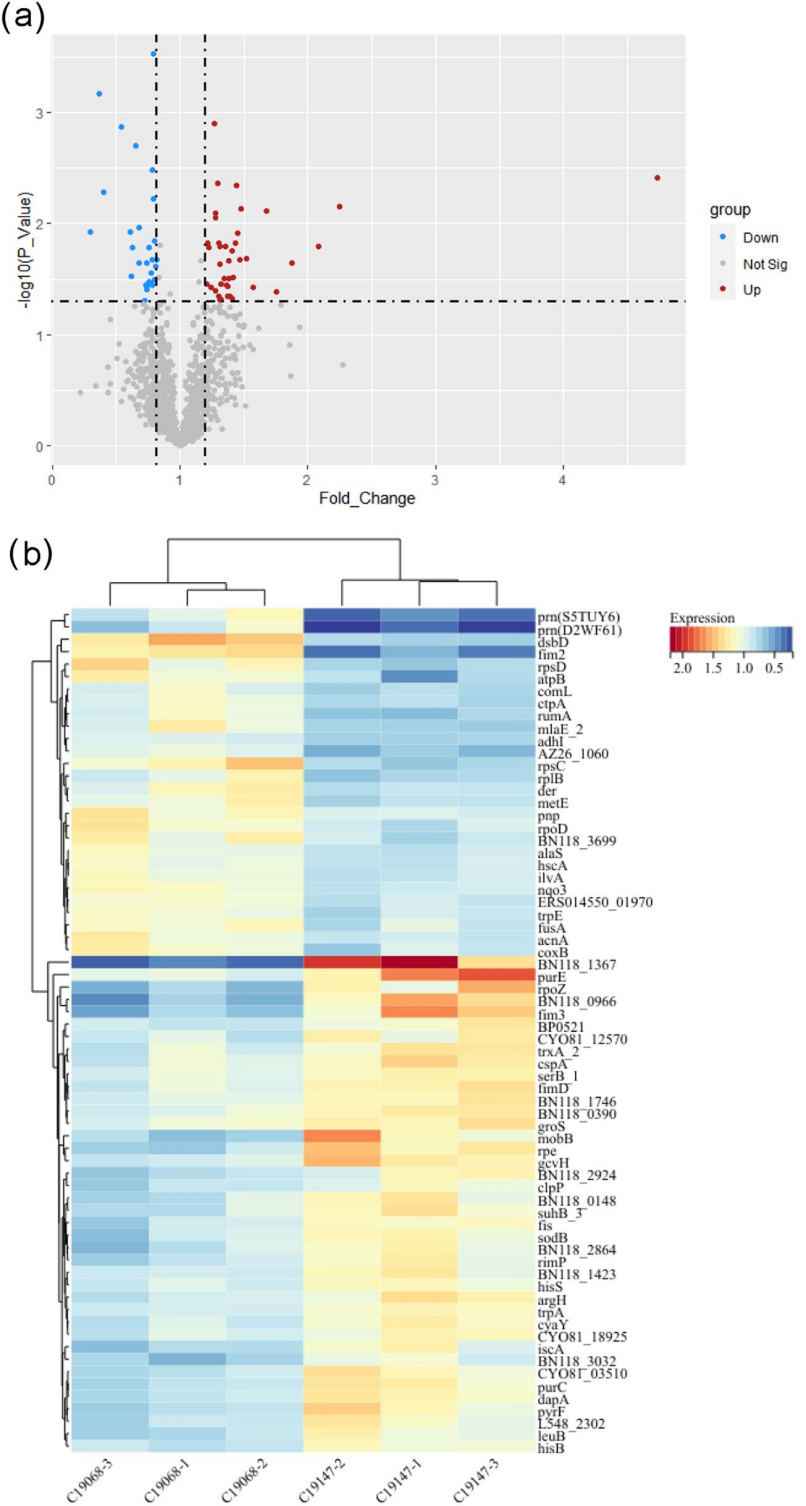
The characteristics of significantly different proteins in whole-cell **a**. Differentially expressed proteins were analyzed by volcano plot between 19,147 (*ptxP1/fhaB3*) and 19,068 (*ptxP3/fhaB1*). **b**. The heat map shows two groups of significantly different proteins

TMT was performed to characterize and compare the whole-cell proteome of 19,147 (*ptxP1/fhaB3*) and 19,068 (*ptxP3/fhaB1*). There are a total of 68 significantly different proteins between the two strains (Supplementary Table 2). Compared with 19,068 (*ptxP3/fhaB1*), 40 proteins in 19,147 (*ptxP1/fhaB3*) are up-regulated and 28 proteins are down-regulated. Differentially expressed proteins were analyzed by volcano plot (Fig. 1a). The heat map shows the clustering of significantly differentially expressed proteins between the two strains (Fig. 1b).

Macrolide resistance is mainly caused by target modifications such as the methylation in a 23S ribosomal RNA (rRNA) adenine residue and the mutation in ribosomal protein L4 or L22 (Fyfe et al. 2016). To explore the key proteins that may be involved, we focused on macrolides-resistant related molecules. Although the Erm, an enzyme that catalyzes A2058 methylation to preclude antibiotic binding, was not detected in the two pertussis strain, the expression of RumA (A0A0N2IMR5) protein was found to be significantly different in the two strains (Fig. 1b). In addition, we found that the expression of ribosomal protein L2, also known as RplB (A0A171JW48), was significantly different in expression between the two strains.

### Function analysis of significantly different proteins in whole-cell

**Fig. 2:**
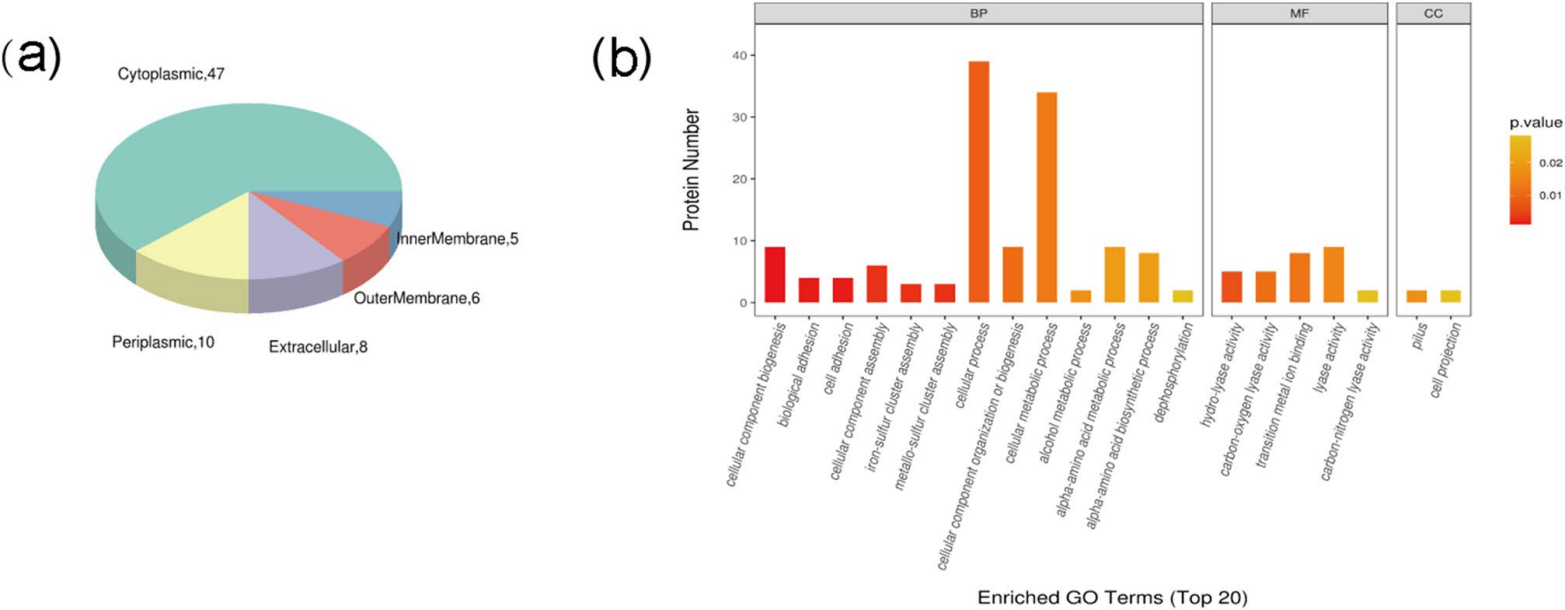
Functional analysis of 19,147 (*ptxP1/fhaB3*) and 19,068 (*ptxP3/fhaB1*) proteins detected in the whole-cell **a**. The subcellular structure of significantly different proteins between the two groups. **b**. The top 20 enriched Gene Ontology (GO) terms of significantly different proteins. *BP* biological process, *MF* molecular function; *CC* cellular component

The outer membrane acts as the first line of defense against the penetration of multiple toxic compounds, including several antimicrobial agents (Munita and Arias 2016). The differential characteristics of outer membrane proteins have been identified in antibiotic-resistant bacteria, which form a specific pattern of antibiotics (Peng et al. 2019). In addition, the loss of some outer membrane proteins reduced the virulence and fitness of bacteria (Smani et al. 2013). Therefore, we further analyzed the subcellular localization of significantly different proteins in the whole-cell (Fig. 2a). There are five kinds of proteins located in the outer membrane, namely FimD (A0A381A3A6), BN118_1423 (A0A0T7CMT4), HscA (Q7VXG7), AZ26_1060 (A0A171JW15), Prn1 (S5TUY6), and Prn1 (D2WF61).

To further analyze the protein characteristics of MR-*Bp*, we performed a GO cluster analysis on the significantly different proteins in the whole-cell proteome. The top 20 enriched GO terms are presented in Fig. 2b. The most noteworthy is the iron–sulfur cluster and metallo-sulfur cluster assembly. The significant differences in metallo-sulfur cluster between the two strains are HscA (Q7VXG7), CyaY (Q7VT96), and IscA (A0A0U0VXP3).

### Protein interaction analysis of the significantly different proteins in whole-cell

**Fig. 3:**
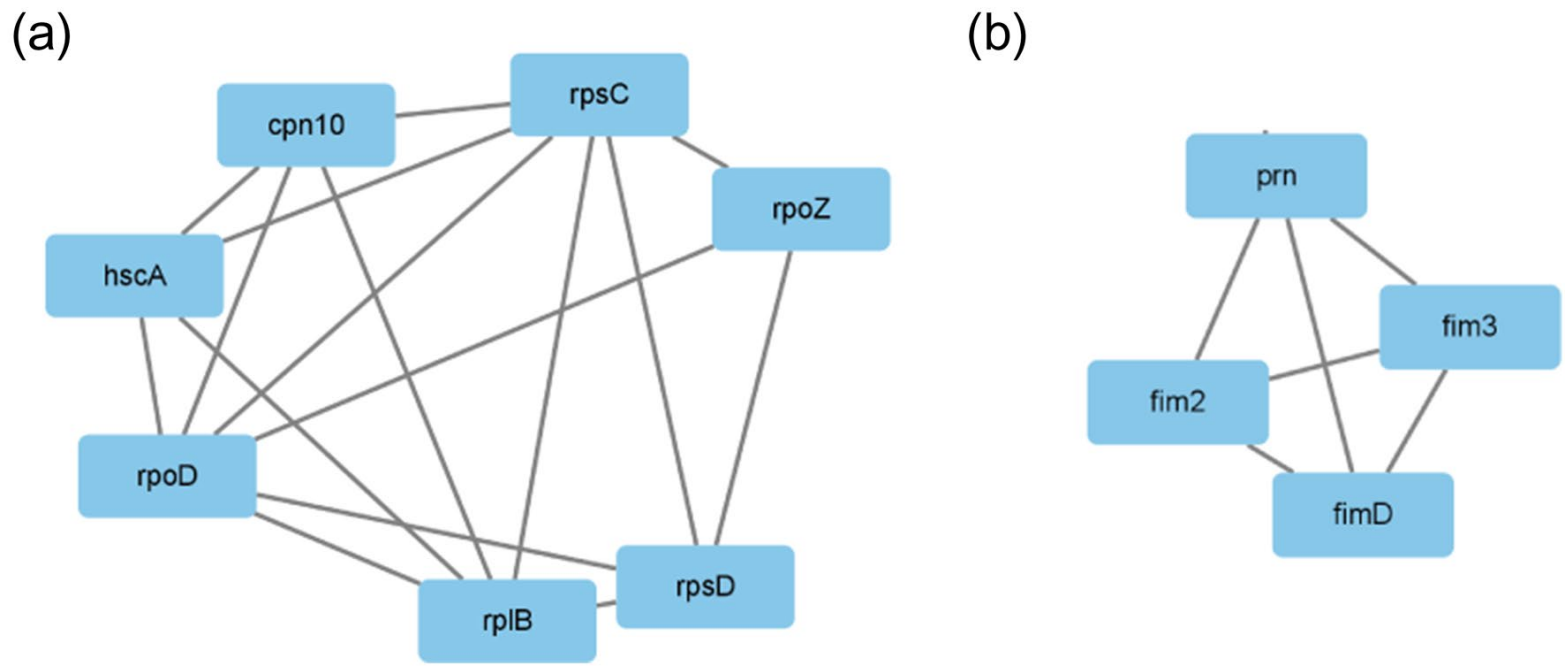
The protein–protein interaction (PPI) network on the significantly different proteins in the whole-cell **a**. The PPI in the most significant module. **b**. The PPI in a second significant module

The functional connection of the significantly different proteins between 19,147 (*ptxP1/fhaB3*) and 19,068 (*ptxP3/fhaB1*) may be a breakthrough to answer the reason for the enhanced resistance and adaptability in MR-*Bp*. The PPI network was constructed using the String website and the figures were generated by Cytoscape (MCODE plug-in). The most significant module (MCODE score = 5.333) contained 7 nodes and 16 edges (Fig. 3a). In the whole-cell proteome in pertussis, Cpn10 (P0A339) and RpoZ (Q7VXZ4) were significantly higher in 19,147 (*ptxP1/fhaB3*) resistant strain compared to 19,068 (*ptxP3/fhaB1*) sensitive strains, and the other five proteins RpsC (Q7VTC7), HscA (Q7VXG7), RpoD (A0A0T7CNP5), RplB (A0A171JW48) and RpsD (P0A4C5) were significantly lower than 19,068 (*ptxP3/fhaB1*) indicating the role of key proteins in the macrolides resistance of *Bp* (Fig. 1b).

The next significant module (MCODE score = 4) contained four genes, which are *prn*, *fim2*, *fim3*, and *fimD* (Fig. 3b). *Prn*, *fim2*, and *fim3* have internationally recognized pertussis virulence genes, and *fim2/fim3* represents distinct serotypes of B. pertussis. In this experiment, compared with 19,068 (*ptxP3/fhaB1*), the expression of Fim3 and FimD increased in 19,147 (*ptxP1/fhaB3*), and the expression of Fim2 and Prn decreased.

### PRM verifies significantly different protein expression levels in whole-cell

To confirm the reliability of the quantitative proteomics analyses, the 11 candidate proteins selected in PPI were evaluated by PRM analyses. The mass spectrometry identification results show that the four target proteins can be accurately identified namely Prn, Fim2, Fim3, and RpoD.

The fold changes of four proteins by PRM are listed in Table 1. Fim2, Prn, and RpoD were down-regulated in 19,147 (*ptxP1/fhaB3*), while Fim3 was up-regulated. The results of the relative quantification demonstrated that the target proteins displayed similar trends between the TMT and PRM analyses, thus supporting the reliability of the proteomics data.

**Table 1:** Comparison of the quantification results between tandem mass tag (TMT) and parallel reaction monitoring (PRM) for the four candidate proteins

Protein name	TMT result			PRM result		
	19,147_average	19,068_average	Ratio_19,147/19068	19,147_average	19,068_average	Ratio_19,147/19068
A0A0T7CNP5(RpoD)	0.9198	1.2183	0.7550	0.0908	0.1262	0.7191
B6DYY0(Fim2)	0.4898	1.3462	0.3639	0.0030	0.5669	0.0053
B6DYY5(Fim3)	1.4421	0.6918	2.0846	2.0524	0.0132	154.9690
D2WF61(Prn)	0.4172	1.0482	0.3980	0.0491	1.1896	0.0412

### The different proteins in whole-cell are related to the biofilm formation

In this study, many differential proteins related to macrolide resistance are closely related to the formation of biofilm, such as metallo-sulfur cluster. Moreover, it has been reported that biofilm-grown *Bp* confers increased tolerance to antimicrobial agents compared with planktonic cultures and biofilm formation provides a fitness advantage to bacteria (Dorji et al. 2016; Deschaine et al. 2018).

**Fig. 4:**
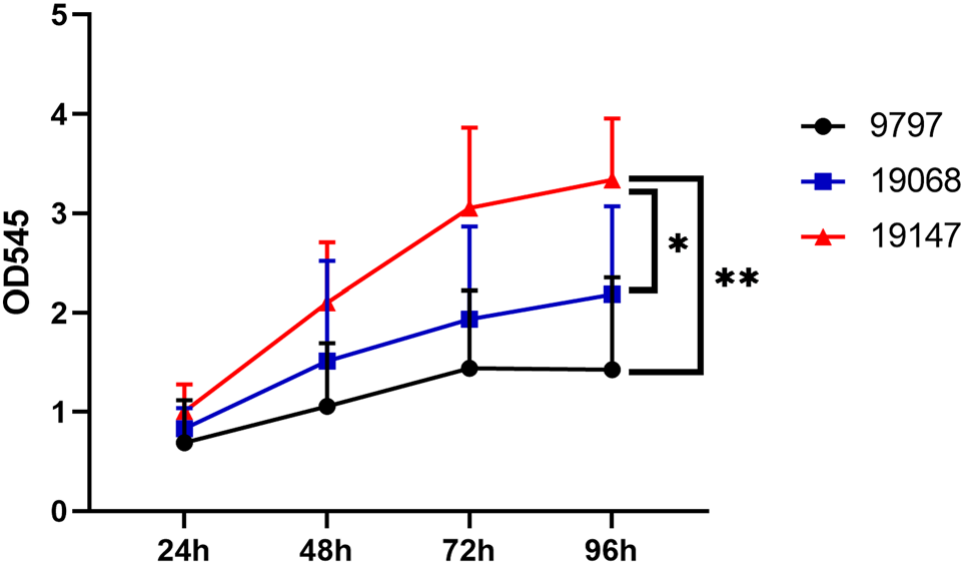
The fhaB3 allele MR-*Bp* increases the formation of biofilm The crystal violet staining revealed the biofilm formation among 19,147 (*ptxP1/fhaB3*), 19,068 (*ptxP3/fhaB1*), and 9797

Crystal violet staining was used to evaluate whether biofilm formation changed in 19,147 (*ptxP1/fhaB3*) and 19,068 (*ptxP3/fhaB1*), of which ATCC strain 9797 was used as the standard strain. Interestingly, crystal violet staining revealed that 19,147 (*ptxP1/fhaB3*) had a significant increase in biofilm formation compared to 19,068 (*ptxP3/fhaB1*) (Fig. 4).

## Discussion

In this study, we determined the significantly different proteins between the dominant lineage of *Bp* strains between China and other countries nearly all over the world through TMT-based proteomics and PRM-based protein quantification. The specific key proteins of MR-*Bp* strain include ribosome-associated proteins (RumA, RplB), outer membrane proteins (FimD, BN118_1423, HscA, AZ26_1060, Prn), Fe/S clusters proteins (HscA, CyaY, IscA), RNA polymerase proteins (RpoD, RpoZ), and virulence proteins (Prn, Fim2, Fim3, FimD). Interestingly, the deletion and mutation of multiple resistance genes encoding these proteins are closely related to the formation of biofilms in other bacterial species. Moreover, the biofilm formation of 19,147 (*ptxP1/fhaB3*) was increased compared with 19,068 (*ptxP3/fhaB1*) (Fig. 4). Based on our limited current knowledge, this is the first report screening the potential mechanisms of macrolides resistance and the fitness of prevalent MR-*Bp* through proteomics.

More and more evidence shows that defects in Fe/S proteins assembly and maturation are closely related to biofilm formation and antibiotic resistance (Mashruwala et al. 2016; Deshpande et al. 2020; Ellepola et al. 2021). Among the three significantly different proteins in the Fe/S cluster, HscA and IscA are involved in the iron–sulfur cluster assembly (Zeng et al. 2007; Mayer 2021), and either hscA mutation or loss of iscA will change the formation of bacterial biofilm (Vasil'eva and Strel'tsova 2013; Rondeau et al. 2019). In the PPI network, RNA polymerase related to the Fe/S cluster contributes to concern, and the RpoZ and RpoD in *Bp* strains may dynamically change macrolides resistance. RpoZ and RpoD, called ω and σ70 factors respectively, are the subunits of the RNA polymerase core enzyme. We found that the RpoZ expression of the 19,147 (*ptxP1/fhaB3*) resistant isolates was increased and the RpoD was decreased correspondingly. Although ω-encoding rpoZ evolves in the ecosystem during colder and drier periods, resulting in an increased rate of biofilm production of rpoZ variants in vitro (Cui et al. 2020), whether it is in Gram-positive or Gram-negative bacteria, defects of rpoZ have been confirmed to impair the ability to completely form biofilms and affect antibiotic resistance (Mukherjee and Chatterji 2008; Weiss et al. 2017; Bhardwaj et al. 2018). Another important function of the ω factor is involved in σ factor recruitment. RpoD mutation is associated with antibiotic resistance (Palace et al. 2020), and we have verified the low expression of RpoD in 19,147 (*ptxP1/fhaB3*) by PRM-based protein quantification.

In the PPI network, except for RNA polymerase (RpoZ and RpoD), both RpsC and RpsD are part of the 30S ribosomal subunit, and their changes will affect antibiotic resistance (Björkman et al. 1999; Gupta et al. 2016). Horizontal gene transfer (HGT) of rplB, also known as ribosomal protein L2, has been shown to play a key role in the genesis of antimicrobial resistance (Gentry and Holmes 2008; Manoharan-Basil et al. 2021). Cpn10, also known as GroES, and over-expression of Cpn10 promote streptomycin resistance (Goltermann et al. 2015).

As we all know, biofilms are communities of microorganisms attached to a surface that is significantly less susceptible to antimicrobial agents than non-adherent planktonic cells (Hall and Mah 2017). It has recently been discovered that biofilm formation was associated with *Bp* resistance. Compared with planktonic cultures, biofilm growing *Bp* conferred increased tolerance to antimicrobial agents (Dorji et al. 2016), which may be a possible reason for the emergence of macrolides resistance of the *ptxP1/fhaB3* strain in China.

After antibiotic resistance, the adaptability of some bacteria will increase rather than decrease, which is usually related to a compensatory mutation in evolution (Durão et al. 2018). The mutation rate of *ptxP1/fhaB3* strain in China is five times than the rest of the global *ptxP1* isolates (Xu et al. 2019). Therefore, the adaptability of the Chinese MR-*Bp* strains may also be produced by compensation mutation, but it needs to be further verified.

Another aspect that deserves attention: the fitness advantage of bacteria is related to biofilm formation. When replication and virulence of drug-resistant bacteria were significantly attenuated, their biofilm formation decreased (Biot et al. 2020). Other studies have reported that hyperbiofilm formation is associated with enhanced virulence traits in *Bp* (Cattelan et al. 2017). The higher biofilm-forming strains will exhibit increased cellular adherence to epithelial cells and contribute to enhanced respiratory tract colonization. FimD (fimbrial subunits), highly expressed in 19,147 (*ptxP1/fhaB3*) strain, is critical in adherence to airway cells and can also serve as specific residues of the chaperone-usher pathway to mediate biofilm formation (Volkan et al. 2013; Guevara et al. 2016). Therefore, the link between the hyperbiofilm-forming ability of *Bp* and enhanced pathogenic phenotypes indirectly suggested that the significant difference in proteins related to biofilm formation may be a crucial factor for the adaptive transmission ability of *ptxP1/fhaB3*/MR-*Bp*.

In addition, the emergence and rapid growth of MR-*Bp* in China may also be related to the following reasons. First, it may be a result of selection pressure from vaccination. Acellular vaccine (ACV) was produced in the 1980s and subsequently replaced the whole-cell vaccine (WCV) in developed countries causing the transition from the *ptxP1* allele to the *ptxP3* allele (Mir-Cros et al. 2019; Safarchi et al. 2016). The replacement of WCV in China occurred a decade later than in developed countries which may be the reason why *ptxP3* strains have not replaced *ptxP1* strains in China (Du et al. 2016). Second, it may also be due to the selection pressure from antibiotics. Before China implemented the strict antibiotic stewardship policies in 2012, the problem of antibiotic abuse in China was very serious (Yin et al. 2013). The overuse of antibiotics has accelerated the rapid emergence of antibiotic resistance. Similarly, Vietnam, which reported *ptxP1/fhaB3* MR-*BP*, also has serious issues with the irrational use of antibiotics (Mao et al. 2015).

However, the limitation of this study is that the proteomics from the culture supernatant was not performed. Besides the proteins from the cells, the outer membrane vesicles (OMVs) from the culture supernatant were also closely attributed to the biofilm formations. Therefore, it is impossible to rule out that some proteins related to the biofilm formations have not been discovered.

## Conclusions

In summary, in the proteomics of 19,147 (*ptxP1/fhaB3*) and 19,068 (*ptxP3/fhaB1*) strains, the significantly different proteins are probably related to biofilm formation. The bacterium growing in biofilm is more resistant to macrolides and contributed to the persistence and transmission, which may be the reason for macrolides resistance and the fitness of *ptxP1/fhaB3* MR-*Bp* strains in China. Further studies focused on biofilms need to be explored much more.

## Supplementary Information

Below is the link to the electronic supplementary material.

Supplementary file1 (XLS 21 KB)

Supplementary file2 (XLS 38 KB)

## Data Availability

The datasets generated during and/or analyzed during the current study are available from the corresponding author on reasonable request.
